# The Current Place of Shock-Wave Lithotripsy for Bile Duct Stones

**DOI:** 10.1155/1990/82957

**Published:** 1990

**Authors:** J. Toouli

**Affiliations:** Gastrointestinal Surgical Unit Flinders Medical Centre Bedford Park South Australia 5042 Australia


assessment of a predictive scoring system, both in patients treated by modern techniques and in a less highly selected group of patients, and the authors indicate that such studies are planned.

Professor John Ham
Department of Surgery
Prince of Wales Hospital
High Street
RANDWICK, NSW 2031
AUSTRALIA

**REFERENCES**

1. Boey, J. H. and Way, L. W. (1980) Acute cholangitis. *Ann. Surg.*, **191**, 264–270.
2. O'Connor, M. J., Schwartz, M. L., McQuarrie, D. G. and Sumner, H. W. (1982) Acute bacterial cholangitis. *Arch. Surg.*, **117**, 437–441.
3. Thompson, J. E., Tompkins, R. K. and Longmire, W. P. (1982). Factors in management of acute cholangitis. *Ann. Surg.*, **195**, 137–145.
4. Leese, T., Neoptolemos, J. P., Baker, A. R. and Carr-Locke, D. L. (1986). Management of acute cholangitis and the impact of endoscopic sphincterotomy. *Br. J. Surg.*, **73**, 988–992.
5. Leung, J. W. C., Chung, S. C. S., Sung, J. J. Y., Banez, V. P. and Li, A. K. C. (1989) *Lancet*, **i**, 1307–1309.

### THE CURRENT PLACE OF SHOCK-WAVE LITHOTRIPSY FOR BILE DUCT STONES

**ABSTRACT**

*Sauerbruch T, Stern M. (1989) Fragmentation of bile duct stones by extracorporeal shock waves: A new approach to biliary calculi after failure of routine endoscopic measures. Gastroenterology, Vol. 96: 146–152*

**A prospective uncontrolled multicenter trial was performed on 113 patients with bile duct stones in whom routine endoscopic approaches for removal of the calculi had failed. These represented 8.3% of the patients referred to the participating centers for endoscopic extraction of the stones. Extracorporeal shock-wave lithotripsy using the Dornier kidney lithotripter achieved stone disintegration in 103 patients (91%). Complete stone clearance from the bile ducts was obtained in 97 patients (86%) after a median of 4 days following extracorporeal shock-wave lithotripsy. Adverse effects, mostly mild, occurred in 36% of the patients. A 30-day mortality rate of 0.9% (in-hospital mortality rate = 1.8%) of this high-risk group with a mean age of 72 yr and a cholangitis rate of 26%, compared favorably with the data given for open surgery. We therefore consider extracorporeal shock-wave lithotripsy a useful method for the treatment of bile duct stones not amenable to routine endoscopic measures**

Reprinted with permission from the authors of the above article from the journal Gastroenterology. Copyright 1989 by the American Gastroenterological Association.

### PAPER DISCUSSION

KEYWORDS: ESWL, choledocholithiasis, endoscopic sphincterotomy

Stones in the common bile duct can be removed surgically or via endoscopy after sphincterotomy.[1,2] In patients with stones in the bile duct after cholecystectomy, the preferred method of treatment is via the endoscopic approach.[3,4] However, in patients with an intact biliary tract, an unresolved question of management is what to do about the stones and the gallbladder once they have been identified at ERCP. In patients who have no contraindications to surgery, the most appropriate course of management is cholecystectomy and choledochotomy for the removal of the bile duct stones.[4] Studies have shown that there is no advantage to clearing the bile duct by endoscopic sphincterotomy prior to cholecystectomy.[5] Furthermore, with modern anaesthesia and surgery, cholecystectomy and choledochotomy is comparable in safety to the endoscopic approach and has the major advantage of removing the diseased gallbladder.[6]

In patients with associated medical illnesses which increase the risks of surgery, it is appropriate for the surgeon to consider the option of treating the bile duct stones via the endoscopic route and leaving the gallbladder in situ.[7] We[8] and others[9] have recently shown that this is an appropriate method of dealing with stones in the bile duct and gallbladder in situ[10] as long as the cystic duct has been demonstrated to be patent at ERCP. If the cystic duct is obstructed, there is a higher incidence of gallbladder complications after the sphincterotomy[8,11] than in patients with a patent cystic duct, hence consideration should be given to cholecystectomy in these patients.

One of the factors which has limited the use of the endoscopic approach for the treatment of stones in the bile duct has been the size of the stones and the presence of a narrowed bile duct distal to the stone.[12] This narrowing is often present in the part of the bile duct which is surrounded by the head of the pancreas. The application of the technique of extracorporeal shockwave lithotripsy (ESWL)[13] to the treatment of these stones opens a new dimension in their treatment particularly in those patients who may have contraindications to a surgical approach. Sauerbruch and Stern report the results of ESWL treatment for a multicentre study done in Germany of 113 patients with bile duct stones in whom endoscopic treatment for removal of stones had failed. These 113 patients represented only 8.3% of patients referred to the various centres for endoscopic treatment and is similar to the experience of other reported series for the failure of extracting large stones. The patients studied had a variety of techniques used in order to extract the stones prior to ESWL. These included attempts at mechanical lithotripsy with a Dornier basket, attempts at dissolution or combination of the two.

In order to carry out ESWL, a nasobiliary catheter was positioned in the bile duct and through the previously performed endoscopic sphincterotomy. The nasobiliary catheter allowed for the installation of radio-opaque contrast medium to visualise the bile duct calculi. A Dornier Kidney Lithotripter HM3 was used to treat the bile duct calculi and most patients (84%) received only one EWSL session. Complete stone clearance from the bile ducts was obtained in 88 (74%) of patients after the first treatment and in 97 (86%) of patients after one or more sessions. Most of the stone fragments passed spontaneously, however some required extraction via the endoscopic route.

Complications attributed to ESWL occurred in approximately 36% of patients, however most of these were minor and did not require treatment. In 27% of patients, mild cardiac arrythmias were noted, however in only one patient did this necessitate cessation of therapy. Transient hemobilia was reported in nine patients, and in six patients septic complications were noted. The septic complications were the most severe and suggest that this form of treatment should be accompanied by appropriate antibiotic cover as bacteremia is common. Emergency surgery was carried out in two patients, one due to septic complications of the gallbladder and the other due to perforation of a juxta papillary diverticulum. The in-hospital plus 30 day mortality was 1.8% for all patients undergoing ESWL therapy.

A factor which is not discussed in detail in the report is the figure that 52 (46%) patients had their gallbladder *in situ* and 39 (35%) had concomitant gallbladder stones. Presumably, in most of these patients the cystic duct was patent, although one of the patients who required emergency surgery after ESWL was as a result of empyema of the gallbladder probably secondary to infection of an obstructed gallbladder. The report does not detail the outcome of the other patients with stones in their gallbladder and how these were treated.

At this stage of its development, the technique of ESWL for the management of large bile duct stones needs to be regarded as an adjunct to existing methods for the management of this condition. The report by Sauerbruch and Stern has illustrated that large stones which cannot be extracted via the endoscopic route, can be shattered "safely" using ESWL, thus promoting their removal. Hence, in patients with stones in the biliary tract after cholecystectomy, this technique further reduces the need for a primary surgical approach to the bile duct. Choledochotomy for bile duct stones will be reserved for the rare instance where endoscopic sphincterotomy plus ESWL and other techniques fail to clear the bile duct. These non-operative methods should be the first line of treatment for all age groups. However, in patients with an intact biliary tract, i.e. gallbladder in situ, the question of whether any form of therapy should be used prior to a surgical approach has not been resolved. This study does not address itself to this question, especially as the follow-up of patients is only brief. However, ESWL provides the technique to ensure that bile ducts may be cleared of calculi even in those patients with large stones or relative narrowing of the distal bile duct.

As illustrated by this study, there is still a failure rate in fragmenting bile duct stones sufficiently so that passage or extraction may result. It is expected that with further refinements of lithotripters and better selection of patients and stones,[14] the failure rate from EWSL will decrease.

Professor J Toouli
Gastrointestinal Surgical Unit
Flinders Medical Centre
Bedford Park
South Australia 5042
AUSTRALIA

### REFERENCES

1. Broughan T.A., Sivak M.V. and Hermann R.E. (1985) The management of retained and recurrent common bile duct stones. *Surgery*, **98**, 748–751.
2. Classen M. and Demling L. (1974) Endosckopischl sphincterotomic der papilli Vateri und stein-extraktion aus dem ductus choledochus. *Dtsch. Med. Wschr.*, **99**, 496–7.
3. Cotton P.B. (1980) Non-operative removal of bile duct stones by duodenoscopic sphincterotomy. *Br. J. Surg.*, **67**, 1–5.
4. Worthley C.S., Watts J. McK. and Toouli J. (1988) Common duct exploration or endoscopic sphincterotomy for choledocholithiasis. *ANZ. J. Surg.*, **59**, 209–216.
5. Neoptolemos J.P., Carr-Locke D.L. and Fossard D.P. (1987) Prospective randomised study of pre-operative endoscopic sphincterotomy versus surgery done for common bile duct stones. *Bri. Med. J.*, **194**, 470–474.
6. Allen B., Shapiro H. and Way L.W. (1981) Management of recurrent and residual common duct stones. *Am. J. Surg.*, **142**, 41–47.
7. Escourrou J., Cordova J.A., Lazorthes F., Frexinos J. and Ribert A. (1984) Early and late complications after endoscopic sphincterotomy for biliary lithiasis with and without gallbladder *"in situ"*. *Gut*, 598–602.
8. Worthley C.S. and Toouli J. (1988) Gallbladder non filling: an indication for cholecystectomy after endoscopic sphincterotomy. *Br. J. Surg.*, **75**, 796–798.
9. Neoptolemos J.P., Carr-Locke D.L., Fraser I. and Fossard D.P. (1984) The management of common bile duct calculi by endoscopic sphincterotomy in patients with gallbladder *in situ*. *Br. J. Surg.* **71**, 69–71.
10. Martin D.F. and Tweedle D.E.F. (1987) Endoscopic management of common duct stones without cholecystectomy. *Br. J. Surg.*, **74**, 209–211.
11. Rosseland A.R. and Solhaug J.H. (1988) Primary endoscopic papillotomy (EPT) in patients with stones in the common bile duct and the gallbladder in situ: a 5–8 year follow-up study. *World. J. Surg.*, **12**, 111–116.
12. Classen M. and Hagenmuller F. (1984) Endoscopic biliary drainage. *Scand. J. Gastroenterology* **19**, (suppl 102) 76–83.
13. Sauerbruch T., Delius M., Paumgartner G., *et al* (1986) Fragmentation of gallstones by extracorporeal shockwaves. *N. Eng. J. Med.* **314**, 818–22.
14. Speer A.G., Webb D.R., Collier N.A., McHutchinson J., St. John D.J.B. and Clunie G.J. (1988) Extracorporeal Shockwave Lithotripsy and the management of common bile duct calculi. *Med. J. Aust.*, **148**, 590–5.

### DOES CHOLANGIOVENOUS REFLUX CAUSE CHOLANGITIS?

### ABSTRACT

*Stewart L, Pellegrini CA, Way LW. Cholangiovenous Reflux Pathways as Defined by Corrosion Casting and Scanning Electron Microscopy. The American Journal of Surgery 1988; 155: 23–8.*

**Using corrosion casting and scanning electron microscopy of the rat biliary tree, we investigated the site and size of the pathways that allow bacteria to reflux from bile to blood. Nonobstructed rat biliary trees were injected retrograde with methylmethacrylate resin at a constant rate of 0.04 ml/min to volumes of 40, 60, 80, 120, 160, and**

